# 5,5′-Bis(benz­yloxy)-2,2′-[hydrazine­diylidenebis(methanylyl­idene)]diphenol

**DOI:** 10.1107/S1600536813030171

**Published:** 2013-11-09

**Authors:** N. R. Sajitha, M. Sithambaresan, M. R. Prathapachandra Kurup

**Affiliations:** a Department of Applied Chemistry, Cochin University of Science and Technology, Kochi 682 022, India; b Department of Chemistry, Faculty of Science, Eastern University, Sri Lanka, Chenkalady, Sri Lanka

## Abstract

The title azine mol­ecule, C_28_H_24_N_2_O_4_, lies about a center of inversion. The dihedral angle between the phenyl ring and the hy­droxy-substituted ring is 70.3 (5)°. The phenolic O–H group forms an intra­molecular hydrogen bond to the azine N atom.

## Related literature

For the biological activity of azines, see: Gul *et al.* (2003 ▶); Kumaraswamy & Vaidya (2005 ▶). For related structures, see: Acrovito *et al.* (1969 ▶); Sithambaresan & Kurup (2011 ▶). For a related synthesis, see: Karmakar *et al.* (2007 ▶).
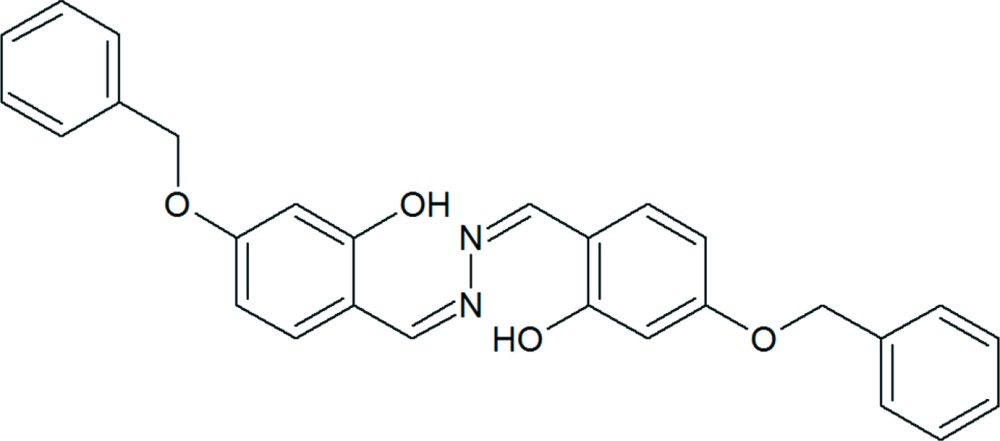


## Experimental

### 

#### Crystal data

C_28_H_24_N_2_O_4_
*M* *_r_* = 452.49
Monoclinic, 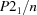

*a* = 12.4748 (17) Å
*b* = 5.3630 (6) Å
*c* = 17.021 (2) Å
β = 90.699 (5)°
*V* = 1138.7 (2) Å^3^
*Z* = 2
Mo *K*α radiation
μ = 0.09 mm^−1^
*T* = 296 K
0.40 × 0.20 × 0.20 mm

#### Data collection

Bruker Kappa APEXII CCD diffractometer
Absorption correction: multi-scan (*SADABS*; Bruker, 2004 ▶) *T*_min_ = 0.965, *T*_max_ = 0.982
6320 measured reflections
2738 independent reflections
1593 reflections with *I* > 2σ(*I*)
*R*_int_ = 0.027

#### Refinement

*R*[*F* ^2^ > 2σ(*F* ^2^)] = 0.044
*wR*(*F* ^2^) = 0.122
*S* = 1.01
2738 reflections
159 parameters
1 restraint
H atoms treated by a mixture of independent and constrained refinement
Δρ_max_ = 0.13 e Å^−3^
Δρ_min_ = −0.15 e Å^−3^

### 

Data collection: *APEX2* (Bruker, 2004 ▶); cell refinement: *SAINT* (Bruker, 2004 ▶); data reduction: *SAINT*; program(s) used to solve structure: *SHELXS97* (Sheldrick, 2008 ▶); program(s) used to refine structure: *SHELXL97* (Sheldrick, 2008 ▶); molecular graphics: *ORTEP-3 for Windows* (Farrugia, 2012 ▶) and *DIAMOND* (Brandenburg, 2010 ▶); software used to prepare material for publication: *SHELXL97* and *publCIF* (Westrip, 2010 ▶).

## Supplementary Material

Crystal structure: contains datablock(s) global, I. DOI: 10.1107/S1600536813030171/ng5346sup1.cif

Structure factors: contains datablock(s) I. DOI: 10.1107/S1600536813030171/ng5346Isup2.hkl

Additional supplementary materials:  crystallographic information; 3D view; checkCIF report

## Figures and Tables

**Table 1:** Hydrogen-bond geometry (Å, °)

*D*—H⋯*A*	*D*—H	H⋯*A*	*D*⋯*A*	*D*—H⋯*A*
O2—H2′⋯N1	0.87 (2)	1.84 (2)	2.625 (2)	148 (2)

**Fig. 1.**
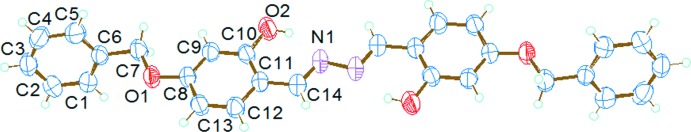
ORTEP view of the title compound drawn with 50% probability displacement ellipsoids for the non-H atoms.

**Fig. 2.**
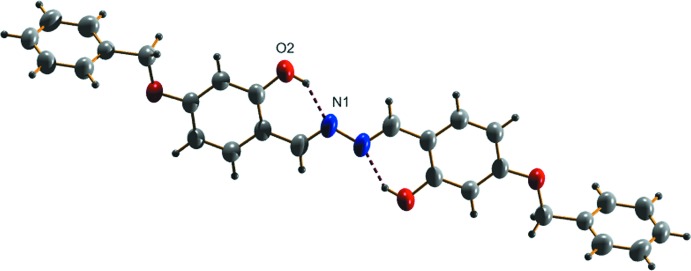
Hydrogen-bonding interactions in the crystal structure of C28H24N2O4.

**Fig. 3.**
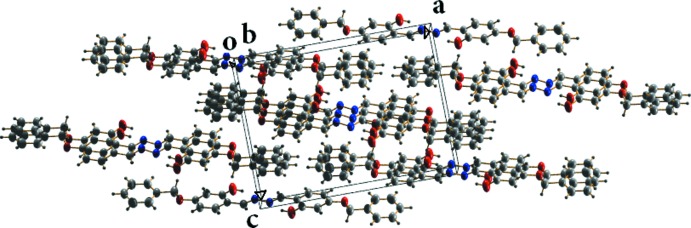
Packing diagram of the compound along the b axis

## supplementary crystallographic information

### 1. Comment

Azines are 2,3-diaza analogous compounds of 1,3-butadiene. Compounds containing azine moiety exhibit a broad spectrum of bilogical activity such as antitumor (Gul *et al.*, 2003), antibacterial (Kumaraswamy & Vaidya, 2005) and many others.

The present compound is analogous to salicylaldehyde azine, in which the fourth position is substituted. The salicyladehyde azine was characterized previously (Acrovito *et al.*, 1969) as luminescent and thermochromic substance in the solid state.

The title compound (Fig. 1) adopts an *E* configuration with respect to C14=N1 (bond distance: 1.288 (21) Å) and is close to the formal C= N bond [1.2758 (19) Å] (Sithambaresan & Kurup, 2011) confirming the azomethine bond formation and the presence of benzylidene moiety. The azomethine and benzaldehyde moieties are nearly planar with interplanar dihedral angle of 2.396 (7)°. The dihedral angle between benzene ring and benzaldehyde ring is 70.329 (46)°. The phenolic O–H forms an intramolecular O–H···N hydrogen bond with a D···A distance of 2.6245 (18) Å (Table 1, Fig. 2). There are very few weak short ring interactions found in the crystal system, but they are not significant to support the network since centriod-centroid distances are above 4 Å. Fig. 3 shows a packing diagram of the title compound viewed along *b* axis.

### 2. Experimental

The title compound was prepared by adapting a reported procedure (Karmakar *et al.*, 2007). 4-Benzyloxy-2-hydroxybenzaldehyde (0.4564 g, 2 mmol) was dissolved in methanol (30 ml). The solution was acidified with 5 drops of glacial acetic acid and hydrazine monohydrate (0.501 g, 1 mmol). The mixture was stirred for 1 h at room temperature. An yellow solution obtained was kept for crystallization for 3 days and the crystalline substance formed was seperated and washed with hexane and dried over P_4_O_10_*in vacuo*. This crystalline substance was recrystallized from dimethylformamide (20 ml) to give yellow needles of 4-benzyloxy-2-hydroxybenzaldehyde azine. The compound was obtained in 65% yield (0.181 g).

IR (KBr, ν in cm^-1^): 1183, 1277, 1213, 1455, 1614, 2945, 3032. ^1^H NMR (400 MHz, DMSO-d_6_, δ in p.p.m.): 8.583 (s, 1H), 5.103 (s, 2H), 7.224 (s, 1H), 7.333–7.446 (m, 5H), 6.585–6.618 (m, 3H).

### 3. Refinement

All H atoms on C were placed in calculated positions, guided by difference maps, with C—H bond distances of 0.93 Å. H atoms were assigned *U*_iso_(H) values of 1.2Ueq(carrier). Omitted owing to bad disagreement were reflections (1 0 1) and (-1 0 1). H atom of O2—H2' bond was located from difference maps and the bond distance is restrained to 0.84±0.02 Å.

### Crystal data

**Table:**

C_28_H_24_N_2_O_4_	*F*(000) = 476
*M**_r_* = 452.49	*D*_x_ = 1.320 Mg m^−^^3^
Monoclinic, *P*2_1_/*n*	Mo *K*α radiation, λ = 0.71073 Å
Hall symbol: -P 2yn	Cell parameters from 1647 reflections
*a* = 12.4748 (17) Å	θ = 3.3–27.3°
*b* = 5.3630 (6) Å	µ = 0.09 mm^−^^1^
*c* = 17.021 (2) Å	*T* = 296 K
β = 90.699 (5)°	Needle, yellow
*V* = 1138.7 (2) Å^3^	0.40 × 0.20 × 0.20 mm
*Z* = 2	

### Data collection

**Table:**

Bruker Kappa APEXII CCD diffractometer	2738 independent reflections
Radiation source: fine-focus sealed tube	1593 reflections with *I* > 2σ(*I*)
Graphite monochromator	*R*_int_ = 0.027
ω and φ scan	θ_max_ = 28.2°, θ_min_ = 2.4°
Absorption correction: multi-scan (*SADABS*; Bruker, 2004)	*h* = −16→14
*T*_min_ = 0.965, *T*_max_ = 0.982	*k* = −7→6
6320 measured reflections	*l* = −22→17

### Refinement

**Table:**

Refinement on *F*^2^	Secondary atom site location: difference Fourier map
Least-squares matrix: full	Hydrogen site location: inferred from neighbouring sites
*R*[*F*^2^ > 2σ(*F*^2^)] = 0.044	H atoms treated by a mixture of independent and constrained refinement
*wR*(*F*^2^) = 0.122	*w* = 1/[σ^2^(*F*_o_^2^) + (0.0436*P*)^2^ + 0.1778*P*] where *P* = (*F*_o_^2^ + 2*F*_c_^2^)/3
*S* = 1.01	(Δ/σ)_max_ < 0.001
2738 reflections	Δρ_max_ = 0.13 e Å^−^^3^
159 parameters	Δρ_min_ = −0.15 e Å^−^^3^
1 restraint	Extinction correction: *SHELXL97* (Sheldrick, 2008), Fc^*^=kFc[1+0.001xFc^2^λ^3^/sin(2θ)]^-1/4^
Primary atom site location: structure-invariant direct methods	Extinction coefficient: 0.0115 (19)

### Special details

**Table:**

Geometry. All e.s.d.'s (except the e.s.d. in the dihedral angle between two l.s. planes) are estimated using the full covariance matrix. The cell e.s.d.'s are taken into account individually in the estimation of e.s.d.'s in distances, angles and torsion angles; correlations between e.s.d.'s in cell parameters are only used when they are defined by crystal symmetry. An approximate (isotropic) treatment of cell e.s.d.'s is used for estimating e.s.d.'s involving l.s. planes.
Refinement. Refinement of *F*^2^ against ALL reflections. The weighted *R*-factor *wR* and goodness of fit *S* are based on *F*^2^, conventional *R*-factors *R* are based on *F*, with *F* set to zero for negative *F*^2^. The threshold expression of *F*^2^ > σ(*F*^2^) is used only for calculating *R*-factors(gt) *etc*. and is not relevant to the choice of reflections for refinement. *R*-factors based on *F*^2^ are statistically about twice as large as those based on *F*, and *R*- factors based on ALL data will be even larger.

### Fractional atomic coordinates and isotropic or equivalent isotropic displacement parameters (Å^2^)

**Table:**

	*x*	*y*	*z*	*U*_iso_*/*U*_eq_	
O1	0.38498 (9)	0.6629 (2)	0.90134 (6)	0.0614 (4)	
O2	0.36761 (13)	0.8385 (2)	0.62715 (7)	0.0803 (4)	
N1	0.47835 (11)	0.5470 (3)	0.53460 (7)	0.0601 (4)	
C1	0.23742 (16)	0.7056 (3)	1.04210 (9)	0.0663 (5)	
H1	0.2071	0.5745	1.0138	0.080*	
C2	0.22037 (16)	0.7219 (4)	1.12153 (10)	0.0699 (5)	
H2	0.1786	0.6028	1.1464	0.084*	
C3	0.26443 (16)	0.9118 (4)	1.16380 (10)	0.0665 (5)	
H3	0.2522	0.9238	1.2175	0.080*	
C4	0.32641 (17)	1.0844 (4)	1.12737 (11)	0.0742 (6)	
H4	0.3573	1.2133	1.1563	0.089*	
C5	0.34358 (16)	1.0684 (3)	1.04737 (11)	0.0706 (5)	
H5	0.3862	1.1868	1.0228	0.085*	
C6	0.29828 (14)	0.8791 (3)	1.00385 (9)	0.0553 (4)	
C7	0.31240 (16)	0.8630 (3)	0.91687 (9)	0.0675 (5)	
H7A	0.2439	0.8320	0.8911	0.081*	
H7B	0.3410	1.0185	0.8970	0.081*	
C8	0.40808 (13)	0.6150 (3)	0.82489 (9)	0.0505 (4)	
C9	0.37165 (14)	0.7540 (3)	0.76205 (9)	0.0565 (4)	
H9	0.3261	0.8887	0.7701	0.068*	
C10	0.40315 (14)	0.6925 (3)	0.68662 (9)	0.0539 (4)	
C11	0.46947 (13)	0.4874 (3)	0.67316 (8)	0.0512 (4)	
C12	0.50273 (14)	0.3498 (3)	0.73856 (10)	0.0611 (5)	
H12	0.5461	0.2110	0.7310	0.073*	
C13	0.47411 (14)	0.4112 (3)	0.81321 (9)	0.0594 (5)	
H13	0.4986	0.3173	0.8557	0.071*	
C14	0.50412 (14)	0.4193 (3)	0.59605 (9)	0.0581 (4)	
H14	0.5466	0.2782	0.5902	0.070*	
H2'	0.3950 (16)	0.782 (4)	0.5836 (10)	0.096 (7)*	

### Atomic displacement parameters (Å^2^)

**Table:**

	*U*^11^	*U*^22^	*U*^33^	*U*^12^	*U*^13^	*U*^23^
O1	0.0707 (8)	0.0746 (8)	0.0390 (6)	0.0179 (6)	0.0081 (5)	0.0004 (5)
O2	0.1141 (12)	0.0833 (9)	0.0435 (7)	0.0295 (8)	0.0038 (7)	0.0089 (6)
N1	0.0613 (9)	0.0802 (10)	0.0388 (7)	−0.0018 (8)	0.0048 (6)	−0.0081 (7)
C1	0.0840 (14)	0.0642 (11)	0.0508 (10)	−0.0055 (10)	−0.0001 (9)	−0.0043 (8)
C2	0.0821 (14)	0.0747 (13)	0.0530 (10)	−0.0032 (10)	0.0090 (9)	0.0074 (9)
C3	0.0769 (13)	0.0786 (13)	0.0441 (9)	0.0174 (11)	0.0023 (9)	−0.0037 (9)
C4	0.0873 (15)	0.0703 (12)	0.0649 (12)	0.0022 (11)	−0.0031 (10)	−0.0198 (10)
C5	0.0802 (14)	0.0650 (12)	0.0670 (12)	−0.0016 (10)	0.0140 (10)	−0.0020 (9)
C6	0.0666 (11)	0.0569 (10)	0.0425 (9)	0.0132 (9)	0.0062 (8)	−0.0006 (7)
C7	0.0836 (14)	0.0724 (12)	0.0466 (10)	0.0215 (10)	0.0098 (9)	0.0025 (8)
C8	0.0510 (9)	0.0604 (10)	0.0403 (8)	−0.0010 (8)	0.0059 (7)	−0.0005 (7)
C9	0.0653 (11)	0.0584 (10)	0.0460 (9)	0.0109 (9)	0.0050 (8)	−0.0003 (8)
C10	0.0617 (11)	0.0589 (10)	0.0409 (8)	−0.0026 (8)	−0.0007 (7)	0.0034 (7)
C11	0.0521 (10)	0.0609 (10)	0.0406 (8)	−0.0033 (8)	0.0040 (7)	−0.0020 (7)
C12	0.0656 (12)	0.0687 (12)	0.0491 (10)	0.0157 (9)	0.0072 (8)	0.0002 (8)
C13	0.0661 (11)	0.0682 (11)	0.0439 (9)	0.0128 (9)	0.0053 (8)	0.0059 (8)
C14	0.0588 (11)	0.0715 (11)	0.0443 (9)	−0.0005 (9)	0.0037 (8)	−0.0057 (8)

### Geometric parameters (Å, º)

**Table:**

O1—C8	1.3607 (17)	C5—H5	0.9300
O1—C7	1.431 (2)	C6—C7	1.496 (2)
O2—C10	1.3505 (19)	C7—H7A	0.9700
O2—H2'	0.874 (15)	C7—H7B	0.9700
N1—C14	1.288 (2)	C8—C9	1.376 (2)
N1—N1^i^	1.396 (2)	C8—C13	1.385 (2)
C1—C6	1.370 (2)	C9—C10	1.387 (2)
C1—C2	1.374 (2)	C9—H9	0.9300
C1—H1	0.9300	C10—C11	1.397 (2)
C2—C3	1.359 (3)	C11—C12	1.394 (2)
C2—H2	0.9300	C11—C14	1.434 (2)
C3—C4	1.360 (3)	C12—C13	1.364 (2)
C3—H3	0.9300	C12—H12	0.9300
C4—C5	1.384 (2)	C13—H13	0.9300
C4—H4	0.9300	C14—H14	0.9300
C5—C6	1.374 (3)		
			
C8—O1—C7	117.41 (12)	O1—C7—H7B	110.1
C10—O2—H2'	107.8 (14)	C6—C7—H7B	110.1
C14—N1—N1^i^	113.47 (19)	H7A—C7—H7B	108.4
C6—C1—C2	121.17 (17)	O1—C8—C9	124.69 (15)
C6—C1—H1	119.4	O1—C8—C13	114.82 (14)
C2—C1—H1	119.4	C9—C8—C13	120.49 (14)
C3—C2—C1	120.14 (18)	C8—C9—C10	119.72 (16)
C3—C2—H2	119.9	C8—C9—H9	120.1
C1—C2—H2	119.9	C10—C9—H9	120.1
C2—C3—C4	119.79 (17)	O2—C10—C9	117.49 (15)
C2—C3—H3	120.1	O2—C10—C11	121.55 (14)
C4—C3—H3	120.1	C9—C10—C11	120.95 (15)
C3—C4—C5	120.15 (18)	C12—C11—C10	117.17 (14)
C3—C4—H4	119.9	C12—C11—C14	120.39 (16)
C5—C4—H4	119.9	C10—C11—C14	122.44 (15)
C6—C5—C4	120.57 (18)	C13—C12—C11	122.50 (16)
C6—C5—H5	119.7	C13—C12—H12	118.8
C4—C5—H5	119.7	C11—C12—H12	118.8
C1—C6—C5	118.16 (16)	C12—C13—C8	119.15 (16)
C1—C6—C7	120.25 (16)	C12—C13—H13	120.4
C5—C6—C7	121.58 (16)	C8—C13—H13	120.4
O1—C7—C6	107.98 (13)	N1—C14—C11	122.21 (17)
O1—C7—H7A	110.1	N1—C14—H14	118.9
C6—C7—H7A	110.1	C11—C14—H14	118.9
			
C6—C1—C2—C3	−0.3 (3)	C8—C9—C10—O2	−178.25 (16)
C1—C2—C3—C4	−0.7 (3)	C8—C9—C10—C11	1.5 (3)
C2—C3—C4—C5	0.7 (3)	O2—C10—C11—C12	179.36 (16)
C3—C4—C5—C6	0.1 (3)	C9—C10—C11—C12	−0.4 (2)
C2—C1—C6—C5	1.1 (3)	O2—C10—C11—C14	0.2 (3)
C2—C1—C6—C7	−177.71 (17)	C9—C10—C11—C14	−179.61 (16)
C4—C5—C6—C1	−1.0 (3)	C10—C11—C12—C13	−0.9 (3)
C4—C5—C6—C7	177.77 (17)	C14—C11—C12—C13	178.29 (17)
C8—O1—C7—C6	179.40 (14)	C11—C12—C13—C8	1.1 (3)
C1—C6—C7—O1	−74.4 (2)	O1—C8—C13—C12	−179.31 (16)
C5—C6—C7—O1	106.85 (19)	C9—C8—C13—C12	0.1 (3)
C7—O1—C8—C9	4.0 (2)	N1^i^—N1—C14—C11	178.83 (17)
C7—O1—C8—C13	−176.67 (16)	C12—C11—C14—N1	−177.39 (17)
O1—C8—C9—C10	177.95 (16)	C10—C11—C14—N1	1.8 (3)
C13—C8—C9—C10	−1.4 (3)		

Symmetry code: (i) −*x*+1, −*y*+1, −*z*+1.

### Hydrogen-bond geometry (Å, º)

**Table:**

*D*—H···*A*	*D*—H	H···*A*	*D*···*A*	*D*—H···*A*
O2—H2′···N1	0.87 (2)	1.84 (2)	2.625 (2)	148 (2)
